# Thematic Review: Ligature Incidents in South West London and St George’s Mental Health NHS Trust Between 1 July 2024 and 30 September 2024

**DOI:** 10.1192/bjo.2026.11578

**Published:** 2026-06-30

**Authors:** Emily Simon Thomas, Robyn-Jenia Wilcha, Rachel Barnsley

**Affiliations:** 1 West London Trust, Crowthorne, United Kingdom; 2 South West London and St George's Mental Health NHS Trust, London, United Kingdom

## Abstract

**Aims:**

The aim was to analyse and identify key themes in all reported “near miss” ligature incidents in South West London and St George's Mental Health NHS Trust between July-September 2024. The hope was to hereby uncover patterns to inform evidence-based changes and to drive improvements in patient safety, standards of care, patient experience and clinical outcomes in the Trust.

**Methods:**

The total number of ligature-related incidents (from Ulysses) was 96 and 83 were included in the review. 13 incidents were excluded due to missing RiO number. Data was collected from RiO progress notes, demographic tab, inclusion records ad risk assessments by three resident psychiatry doctors. Data collected included demographic details, diagnosis, location of incident, level of observations, mental state at the time of the incident, ligature method, Mental Health Act status, substance misuse, number of previous admissions, and previous ligature incidents. The data collection tool was piloted and areas of unclarity discussed by all three data collectors and consensus reached.

**Results:**

Diagnostically, neurodevelopmental conditions were present in 65.1%, Emotionally Unstable Personality Disorder (EUPD) diagnosis in 53.0%, depression in 34.9%. In terms of other clinical risk factors: 89.2% had multiple psychiatric diagnoses, 73.5% were detained under the Mental Health Act (MHA), the mean number of psychiatric admissions: 4 ± 4, 51.8% were noted as aggressive, alcohol misuse was a factor for 18.1%, drug misuse for 15.7%. Looking at self harm history, 95.2% had a current history of deliberate self-harm (DSH), 98.8% had a historical risk of DSH, 94.0% had previously tied a ligature, and 97.6% had used alternative methods of self-harm. Social and Demographic Factors: 85.5% were unmarried, 79.5% were unemployed, 49.4% were on long-term sick leave, 24.1% lived alone, and 1.2% were homeless.

**Conclusion:**

Thematically, there was notable over-representation of women and girls in reported incidents. nearly two-thirds of incidents involved individuals with a neurodevelopmental diagnosis (e.g., ASD, ADHD), and around half had an EUPD diagnosis.

Recommendations:

1. Improve Incident Documentation: develop clear guidelines to help staff include critical details.

2. Address Observations-Related Incidents: Reinforce active engagement and proximity. Observing staff to conduct environmental review including blind spots and support with co-developed shared ligature care plans which are regularly reviewed.

3. Strengthen Environmental Safety: Continue minimising ligature points, securing hazardous items (BP machine cables).

4. Support for Neurodevelopmental Diagnoses: provide targeted training for staff.

5. Support for EUPD: promoting DBT-informed approaches on wards.

7. Involving Patients in Next Steps: engage service users in future planning and prevention strategies.

8. Providing Holistic Debriefing for both patients and staff impacted by these incidents.

This work was presented in the Trust Learning and Improvement Group and an improvement plan developed.

